# Differential Regulatory Effects of Cannabinoids and Vitamin E Analogs on Cellular Lipid Homeostasis and Inflammation in Human Macrophages

**DOI:** 10.3390/antiox15010119

**Published:** 2026-01-16

**Authors:** Mengrui Li, Sapna Deo, Sylvia Daunert, Jean-Marc Zingg

**Affiliations:** 1 Department of Biochemistry and Molecular Biology, Miller School of Medicine, University of Miami, Miami, FL 33136-6129, USA; mxl1317@miami.edu (M.L.); sdeo@med.miami.edu (S.D.); 2 Dr. John T. Macdonald Foundation Biomedical Nanotechnology Institute, University of Miami, Miami, FL 33146-2101, USA; 3 University of Miami Clinical and Translational Science Institute, University of Miami, Miami, FL 33136-6129, USA

**Keywords:** macrophages, cannabinoids, vitamin E acetate, inflammation, lipid homeostasis

## Abstract

Cannabinoids can bind to several cannabinoid receptors and modulate cellular signaling and gene expression relevant to inflammation and lipid homeostasis. Likewise, several vitamin E analogs can modulate inflammatory signaling and foam cell formation in macrophages by antioxidant and non-antioxidant mechanisms. We analyzed the regulatory effects on the expression of genes involved in cellular lipid homeostasis (e.g., CD36/FAT cluster of differentiation/fatty acid transporter and scavenger receptor SR-B1) and inflammation (e.g., inflammatory cytokines, TNFα, IL1β) by cannabinoids (cannabidiol (CBD) and Δ9-tetrahydrocannabinol (THC)) in human THP-1 macrophages with/without co-treatment with natural alpha-tocopherol (*RRR*-αT), natural *RRR*-αTA (αTAn), and synthetic racemic *all*-*rac*-αTA (αTAr). In general, αTAr inhibited both lipid accumulation and the inflammatory response (TNFα, IL6, IL1β) more efficiently compared to αTAn. Our results suggest that induction of CD36/FAT mRNA expression after treatment with THC can be prevented, albeit incompletely, by αTA (either αTAn or αTAr) or CBD. A similar response pattern was observed with genes involved in lipid efflux (ABCA1, less with SR-B1), suggesting an imbalance between uptake, metabolism, and efflux of lipids/αTA, increasing macrophage foam cell formation. THC increased reactive oxygen species (ROS), and co-treatment with αTAn or αTAr only partially prevented this. To study the mechanisms by which inflammatory and lipid-related genes are modulated, HEK293 cells overexpressing cannabinoid receptors (CB1 or TRPV-1) were transfected with luciferase reporter plasmids containing the human CD36 promoter or response elements for transcription factors involved in its regulation (e.g., LXR and NFκB). In cells overexpressing CB1, we observed activation of NFκB by THC that was inhibited by αTAr.

## 1. Introduction

After an inflammatory stimulus, infection, or injury, monocytes differentiate into macrophages, which are able to phagocytose pathogens, modified proteins, and debris from damaged and apoptotic cells. The accumulation of modified proteins and lipids exceeding the macrophages’ storing and handling capacities is considered a main driver of the chronic inflammatory response and is found in many diseases, including atherosclerosis, steatohepatitis, obesity-induced insulin resistance, and neurodegeneration. Dysregulation of lipid homeostasis leads to increased production of free radicals, inflammation, and lipotoxicity, resulting in macrophage foam cell formation and cardiovascular, metabolic, and liver disease under sterile conditions (without bacterial infection) [1,2].

Vitamin E (alpha-tocopherol, αT) is the main natural antioxidant in the lipid phase with important regulatory functions in inflammation and cellular lipid metabolism. By scavenging free radicals, vitamin E can prevent lipid peroxidation that may occur as a result of metabolic diseases associated with excess lipids and can reduce inflammation. In recent years, other forms of vitamin E have gained attention for their superior anti-inflammatory properties compared to αT, leading to a shift in research focus. In macrophages, several studies have shown differential anti-inflammatory effects of different vitamin E analogs (α-, β-, γ-, δ-tocopherol, tocotrienols) and derivatives (α-tocopheryl phosphate, αTP) as a result of their regulatory effects on signal transduction and gene expression that can occur by antioxidant and non-antioxidant molecular mechanisms [3,4]. A few receptors have been identified with potential binding sites for vitamin E analogs in their protein structure (e.g., 67 kDa laminin receptor, CD36 scavenger receptor, cannabinoid receptors 1 and 2 (CB1, CB2)), but whether and how the binding of vitamin E affects their activity is often not investigated in detail [5,6,7]. Similar to vitamin E, cannabinoids can modulate inflammation and lipid homeostasis in macrophages by regulating cannabinoid receptors (CB1, CB2). However, the molecular mechanisms by which vitamin E analogs and cannabinoids modulate chronic inflammation are not clear and may also involve other receptors, such as transient receptor potential of vanilloid subtype 1 (TRPV-1) or G protein-coupled receptor 55 (GPR55).

Cannabinoid receptors play a role in lipid/energy homeostasis and are considered important pharmacotherapeutic targets in metabolic disease, inflammation, and cancer-related anorexia-cachexia [8,9,10]. The plant-derived cannabinoids Δ9-tetrahydrocannabinol (THC) and cannabidiol (CBD) can bind to the two main receptors (CB1, CB2) that trigger cellular signaling important for lipid homeostasis, inflammation, and cancer. CB1 activation is proatherogenic by promoting inflammation and oxidative stress that can cause endothelial dysfunction and atherosclerosis, whereas CB2 activation is anti-atherogenic [11]. Accordingly, a recent cross-sectional study reported an increased risk for early development of vascular disease in subjects with chronic cannabis smoking and THC ingestion [12]. The transient receptor (TRPV-1) has also been implicated in the regulatory effects of cannabinoids. TRPV-1 is a thermal nociceptor and plays an important role in the detection of painful stimuli such as heat, acid, and irritant chemicals. The TRPV-1 receptor is not just involved in inflammation and autoimmune disease but also in cancer development. The regulatory involvement of TRPV-1 is complicated by the multiple and often contradictory pro-inflammatory/anti-inflammatory and pro-cancerogenic/antineoplastic actions described for this receptor.

Several vitamin E analogs have been previously investigated by our and other laboratories for their ability to regulate lipid accumulation in monocytes/macrophages [13,14,15] and pre-adipocytes/adipocytes [16]. However, the molecular mechanisms by which increased accumulation of lipids may lead to increased inflammation are not completely resolved and may involve regulatory effects on signal transduction (e.g., PI3K/Akt) and gene expression (e.g., via peroxisome proliferator-activated receptor gamma, PPARγ) [17,18,19]. In several studies, we and others have found that vitamin E (alpha-tocopherol, αT) and some of its analogs reduced oxLDL uptake and lipid accumulation by reducing surface exposure and signaling of the CD36/FAT scavenger receptor/fatty acid transporter [13,14,15,20,21,22]. In studies using stabilized vitamin E analogs esterified at the chromanol hydroxy group, such as alpha-tocopheryl acetate (αTA), it is commonly assumed that they act as a precursor of αT since they are efficiently hydrolyzed to αT by pancreatic and intestinal esterases during intake and digestion [23]. However, hydrolysis may not always be complete, as suggested by reported differences in bioavailability of αT and αTA in humans and animals [24,25]. Situations in which αTA is not completely hydrolyzed may occur in skin, lungs, and intestine, e.g., in the absence of sufficient dietary fat [26,27,28,29,30,31,32,33,34,35,36]. Since the chromanol hydroxy group is blocked in αTA, the intact form is not able to act directly as a chemical antioxidant and may exert non-antioxidant regulatory effects on signaling and gene expression similar to other modified forms of vitamin E [13,19,37,38,39].

The CD36/FAT scavenger receptor may represent a potential link between cellular fatty acid accumulation, inflammatory signaling, cannabinoids, and vitamin E. Compelling evidence shows that CD36/FAT is a sensor and regulator of fatty acid import and energy homeostasis in several cell types, including macrophages [40]. Together with a related receptor, the scavenger receptor B1 (SR-B1), CD36/FAT also mediates the transport of vitamin E and its analogs into cells and across the intestinal epithelium and the blood–brain barrier [13,41,42]. Together with Toll-like receptors, CD36/FAT in macrophages is also the receptor for several pathogens and modified molecules and is thought to be at the beginning of the inflammatory response, including the sterile inflammatory response after exposure to cholesterol crystals [43]. Interestingly, in human blood, peripheral blood mononuclear cells’ CD36/FAT expression is upregulated by natural and synthetic agonists of CB1 and CB2 (e.g., arvanil, anandamide, Win55,212-2), involving activation of PPARγ [44,45] and may involve intracellular transport by fatty acid binding proteins 4 and 5 (FABP4/aP2, FABP5) [46,47,48,49]. Accordingly, selective inhibition of CB1 by rimonabant or AM251 reduced lipid accumulation and foam cell formation [45,50]. In contrast, selective activation of CB2 by JWH-015 reduced foam cell formation and the inflammatory response, effects that are inhibited by the selective CB2 antagonist SR144528 [51,52]. Thus, the modulation of CD36/FAT by cannabinoids and vitamin E analogs (and, as investigated here, by αTA) may affect a number of signal transduction and gene expression pathways relevant to inflammation and lipid homeostasis [53]. Accordingly, a relationship between CD36/FAT regulation, vitamin E deficiencies, and diseases with aberrant lipid/energy homeostasis, such as atherosclerosis, obesity, liver disease, and cancer, has been identified [54,55,56].

Recently, it was reported that vitamin E acetate is able to bind to cannabinoid receptors (CB1, CB2) and inhibit the binding of THC (>50% reduction) [5,6]. However, the molecular mechanisms by which cannabinoids and vitamin E analogs may affect signaling and the expression of genes relevant to inflammation and lipid accumulation in macrophages have not been elucidated. Therefore, in this study, we characterized and compared the molecular regulatory effects of cannabinoids and vitamin E analogs (αTAn, αTAr) on the expression of genes relevant to cellular lipid homeostasis that may contribute to the increase in the level of lipids, free radicals, and inflammatory cytokines observed in inflammatory and metabolic diseases.

## 2. Materials and Methods

### 2.1. Source of Compounds, Vitamins, Cannabinoids

*RRR*-α-tocopherol (αT) was dissolved in ethanol as a 50 mM stock solution. Natural *RRR*-alpha-tocopheryl acetate (αTAn) and synthetic *all-rac*-alpha-tocopheryl acetate (αTAr) were purchased from Sigma (Darmstadt, Germany) and dissolved in ethanol as 50 mM stocks (Figure S1). The cannabinoids Δ9-tetrahydrocannabinol (THC) and cannabidiol (CBD) were purchased from Sigma and dissolved in ethanol as 6 mM stock. The CD36 inhibitor sulfo-N-succinimidyl oleate (SSO) and the NF*κ*B inhibitor JSH-23 were purchased from Sigma and dissolved in DMSO.

### 2.2. Cell Culture

The human THP-1 acute monocytic leukemia cell line (THP-1, ATCC TIB-202) was grown in RPMI/10% FBS, 2 mmol/L L-glutamine, 1.0 mmol/L sodium pyruvate, 4.5 g/L glucose, and 100 μg/mL streptomycin [15]. THP-1 cells are an established model to study regulatory mechanisms involved in inflammation and foam cell formation in monocytes/macrophages [57,58]. THP-1 monocytes were differentiated into macrophages with phorbol 12-myristate 13-acetate (PMA) (100 nM) for 48 h, which is known to lead to differentiated macrophages with maximal expression of CD36/FAT [59,60,61], and then treated with vitamin E analogs (*RRR*-alpha-tocopherol (αT), natural *RRR*-alpha-tocopheryl acetate (αTAn), synthetic *all-rac*-alpha-tocopheryl acetate (αTAr), 50 μM final), and cannabidiol (CBD) or Δ9-tetrahydrocannabinol (THC), 6 μM final) for an additional 18–24 h [57]. The human HEK293 human embryonic kidney cells (ATCC CRL-3216) were grown in DMEM/10% FBS and 1% streptomycin. and then treated with vitamin E analogs (*RRR*-alpha-tocopherol (αT), natural *RRR*-alpha-tocopheryl acetate (αTAn), synthetic *all-rac*-alpha-tocopheryl acetate (αTAr), and cannabidiol (CBD), Δ9-tetrahydrocannabinol (THC)) for an additional 18–24 h. HEK-TRPV-1 cells (hTRPV-1-HEK, #CT6105, Charles River Laboratories, Cleveland, OH, USA) were grown in DMEM/10% FBS, 1% MEM amino acids solution (50×), and 1% streptomycin. HEK-CB1 cells (EIU005, Kerafast, Boston, MA, USA) were grown in DMEM/10% FBS, 1% geneticin selective antibiotic, and 1% streptomycin.

### 2.3. Cell Proliferation and Toxicity Assay

Cells were plated into 96-well microtiter plates, grown for 24 h, and then treated as indicated for 24 h, 48 h, or 72 h (αT, αTAr, αTAn (50 μM); THC, CBD (6 μM)). The cytotoxic effects of cannabidiol (CBD) and Δ9-tetrahydrocannabinol (THC) were measured by quantifying the release of lactate dehydrogenase (LDH) from the THP-1 macrophages. The supernatants of cultured cells, 150 μL per well in a 96-well plate, were collected into microtiter plates for LDH measurements (CytoTox 96^®^, Promega, Madison, WI, USA). Then, reagents for the LDH assay were added (50 μL per well) and incubated at 37 °C for 30 min. A total assay volume of 50 μL was made up with 1× stop buffer and then measured at 492 nm using a microtiter plate reader (Clariostar, BMG Labtech, Cary, NC, USA). The average values of the culture medium background were subtracted from all values of experimental wells.

### 2.4. Foam Cell Formation Assay Using Oil Red O and Nile Red Staining

Lipid accumulation in cultured cells by staining with Oil Red O has been commonly used macroscopically to visualize adipose cell colonies from cultures of pre-adipose cell lines. The determination of lipids with Oil Red O can be used for any in vitro cultured cells that may accumulate triglycerides or cholesteryl esters, whether they are adipocytes or nonadipocytes [62]. THP-1 macrophages were seeded into 6-well plates at 2 × 10^5^ cells/mL and treated as described above. Cells were washed with 2 mL PBS twice [22]. Subsequently, cells were stained with Oil Red O staining solution for 90 min, rinsed extensively with water, and fixed with 1 mL 10% formaldehyde/PBS (Thermo Scientific Chemicals, Waltham, MA, USA) for 1 h at room temperature. After discarding the fixation solution, the cells were washed twice with water and extracted with ethanol. The amount of the Oil Red O staining was quantified using a microtiter plate reader (Clariostar, BMG Labtech). The relative signals were normalized as the fold induction over untreated controls, and each assay was performed in triplicate.

For staining of lipids with Nile Red, THP-1 macrophages were grown and treated as above, washed, and fixed with 10% formaldehyde in PBS for one hour [22,63]. The cells were washed one more time with PBS and then stained with Nile red (1/100 dilution of 100 μg/mL stock solution of Nile Red in acetone) and then kept in the dark for 20 min. Macrophages were washed three times with PBS and fixed with 10% formaldehyde/PBS. The cell fluorescence reflecting neutral lipids was measured using a Beckman Coulter Cytoflex S FACS (ex561/em610, 10,000 cells) (Beckman Coulter Inc., Brea, CA, USA), and the median ± standard deviation (SD) was calculated relative to the untreated control.

### 2.5. Cell-Surface CD36 Assessment by Fluorescence-Activated Cell Sorting (FACS)

THP-1 cells (10^6^ cells in a 6-well plate) were treated as indicated for 6 h, harvested, and the presence of CD36 on the cell surface was analyzed by FACS as previously described using a monoclonal anti-CD36–FITC antibody (SMO, Ancell, Bayport, MN, USA) and subsequently processed for statistical analysis [20].

### 2.6. Isolation of RNA, cDNA Synthesis, and RT-qPCR Assay

Cultured THP-1 macrophages were treated as described above. Experimental RNA was extracted and isolated from THP-1 macrophages using TRIzol™ Reagent (Invitrogen, Carlsbad, CA, USA). The RNA concentration and purity were measured by the A260/A280 ratio. cDNA was synthesized using a high-capacity cDNA reverse transcription kit (Applied Biosystems, Waltham, MA, USA) and used for the RT-qPCR assay. PCR for cDNA was performed using Taqman Master Mix (Thermo Fisher, Waltham, MA, USA) and Taqman gene expression probes (Table S1), with 10 s 98 °C, 10 s 60 °C, 10 s 72 °C, 4 min 72 °C for 40 cycles. Quantification PCR was performed using Taqman probes and a QuantStudio 5 Real-Time PCR system (Thermo Fisher Scientific, Waltham, MA, USA). Relative mRNA expression levels were calculated using ∆CT values and GAPDH (glyceraldehyde-3-phosphate dehydrogenase) as internal control.

### 2.7. Cytokine Secretion by ELISA

Measurement of cytokine levels has yielded useful information on the pathologic process in different disease states, and it may also be of use in the monitoring of disease progression and/or inflammation. The detection of secreted cytokine protein is by far the most widely used type of analysis. Secreted cytokines are typically measured by ELISA or radioimmunoassay due to the simplicity and sensitivity of these methods [64]. The supernatants were prepared from cultured cells after each treatment, and TNFα (ab100654, Abcam Co., Ltd., Waltham, MA, USA), IL1β (ab217608, Abcam), and IL6 (ab178013, Abcam) were detected by ELISA.

### 2.8. Measurement of Reactive Oxygen Species (ROS) by Flow Cytometric Analysis

THP-1 macrophages were treated with THC, CBD (6 μM), αTAn, and αTAr (50 μM) for 18 h, and then the level of intracellular ROS was assessed with probe 2′,7′-dichlorofluorescein diacetate (DCFH-DA) (Thermo Fischer Scientific), which oxidizes to fluorescent dichlorofluorescein (DCF) in the presence of ROS. Briefly, THP-1 macrophages were scraped and suspended in FBS-free RPMI 1640 at a given concentration of 2 × 10^5^ cells/mL, and 1 mL of cell suspension was incubated in 5 μM DCFH-DA for 30 min in darkness. After being washed twice with PBS and fixed with 1 mL 10% formaldehyde/PBS (Thermo Scientific Chemicals), the mean fluorescence intensity was determined and analyzed using a flow cytometer (Beckman Coulter Cytoflex S FACS) at an excitation wavelength of 492 nm and an emission wavelength of 517 nm [22,65].

### 2.9. Plasmids

The plasmid pCD36extprobasic, containing a 4557 bp promoter sequence in front of the firefly luciferase reporter gene, has been previously described [66]. The LXR reporter plasmid pLXR-RE was kindly provided by Dr. K. Griffett and Dr. T. P. Burris, Saint Louis University, Saint Louis, MO, USA, and has been previously described [67]. The NFκB reporter plasmid pNFκB-RE was from Clontech (San Jose, CA, USA). The Nrf2 reporter plasmid pNrf2-RE was kindly provided by Dr. L. Villacorta, Morehouse School of Medicine, Atlanta, GA, and has been previously described [66]. The reporter plasmid for PPARγ (PPARγ-RE, pDR1) has been previously described [68].

### 2.10. Transfection and Dual Luciferase Assay

HEK293, HEK-TRPV-1, and HEK-CB1 cells were transfected using ViaFect™ Transfection Reagent according to the manufacturer’s protocol (Promega E4981). Cells (1.5 × 10^5^ cells/well) were seeded in 6-well plates 24 h before transfection. Plasmids pRL-TK (Promega) and specific promoter reporter vectors (pCD36extprobasic, pLXR-RE, pNrf2-RE, pNFκB-RE, pPPARγ-RE (Figure S2)) were mixed in DMEM and incubated with transfection reagent for 20 min at room temperature. Transfection complexes were then gently added to individual wells of the 6-well plate. Three hours after transfection, cells were treated with vitamin E analogs and/or THC/CBD. The cells were harvested with passive lysis buffer (PLB) and measured following the dual luciferase assay protocol provided by the manufacturer and using a Clariostar microtiter plate reader (BMG Labtech). The relative signals were normalized as the fold induction relative to unstimulated controls. Each assay was performed in triplicate.

### 2.11. Statistical Analysis

All data are expressed as mean ± standard error of the mean (SEM) and were analyzed using GraphPad Prism version 10. All experiments were performed with independent replicates at least 3 times. Statistical significance was determined using a two-tailed Student’s *t*-test for comparisons between two groups and two-way ANOVA for multiple-group comparisons.

## 3. Results

### 3.1. THC Increases Lipid Accumulation in THP-1 Macrophages, and Vitamin E or CBD Can Prevent It

**Figure 1:**
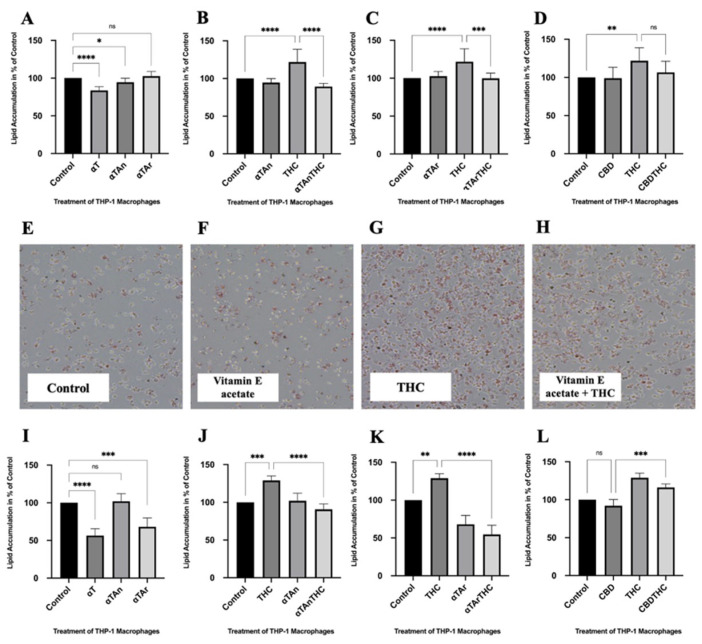
**Lipid accumulation in THP-1 macrophages.** Cells were treated as indicated for 24 h (αT, αTAr, αTAn (50 μM); THC, CBD (6 μM)). (**A**–**D**) Staining was performed with Oil Red O, extraction with isopropanol, and absorbance was measured at OD 500 nm. An inhibitory effect was observed in THP-1 macrophages after treatment with αT, αTAr, and αTAn. (**B**) Stronger lipid accumulation was observed with THC, which was significantly inhibited with co-treatment with αTAn. (**C**) Significant inhibitory effects of αTAr were observed after THC co-treatment. (**D**). Stronger staining was observed with THC, which was non-significantly inhibited by co-treatment with CBD. (**E**–**H**) Staining was performed with Oil Red O in THP-1 macrophages and analyzed by light microscopy. THC induced more lipid droplets inside macrophages, and vitamin E acetate showed an inhibitory effect on lipid accumulation. 10× magnification. (**I**–**L**). Staining with Nile Red, extraction with isopropanol, and measurement by FACS at ex561/em610 nm. (**E**) A weak inhibitory effect was observed in THP-1 macrophages treated with αT, αTAr, and αTAn. (**F**) Stronger staining was induced by THC, which was significantly inhibited with co-treatment with αTAn. (**G**) Significant inhibitory effects were observed after αTAr and THC co-treatment, and αTAr had slightly stronger inhibitory effects than αTAn. (**H**) Stronger staining was observed with THC, which was significantly inhibited by co-treatment with CBD. (mean ± SEM, n = 6 for THC, CBD, αT, αTAr, αTAn, * *p* < 0.05, ** *p* < 0.005, *** *p* < 0.0005, **** *p* < 0.0001 relative to untreated control set to 100%; ns: non-significant).

To unravel the regulatory effects of cannabinoids and vitamin E on lipid accumulation in macrophages, we differentiated human THP-1 monocytes into macrophages and treated them with cannabinoids THC or CBD (each 6 μM) with and without co-treatment with either αT, αTAn, or αTAr (each 50 μM) for 18 h. Similar concentrations of THC and CBD were previously used to study regulatory effects on cytokines in THP-1 macrophages [69]. For intact αTAn and αTAr, such elevated concentrations are unlikely to be physiologically relevant except under specific conditions, such as direct exposure of epithelial cells to vapors or supplements (e.g., lungs, intestines, nasal passages, eyes), topical application (e.g., lotions, creams), or administration via direct injection into tissues (e.g., as adjuvants). To measure the effects of these treatments on lipid homeostasis, we measured the accumulation of lipids by Oil Red O staining and measured the absorbance after extraction. Regulatory effects with all forms of vitamin E alone (αT, αTAn, and αTAr) were weak and only significantly inhibitory for αT and αTAn (Figure 1A). Δ9-tetrahydrocannabinol (THC)-treated THP-1 macrophages showed significantly higher lipid accumulation than control cells, which was significantly inhibited by αTAn (Figure 1B) and αTAr (Figure 1C). Similarly to vitamin E, treatment with CBD reduced lipid accumulation after THC treatment, but the effect was non-significant (Figure 1D). In contrast to THC, CBD treatment did not increase lipid accumulation. Increased lipid accumulation induced by THC treatment was also observable with light microscopy after Oil Red O staining, but the inhibitory effects were less evident using this method (Figure 1E–H). Similar but more pronounced results were obtained when cells were stained with Nile Red and analyzed by FACS (Figure 1I–L). In general, αTAr showed greater inhibitory activity compared with αTAn. No significant effects on cell cycle progression or cytotoxicity were detected under these treatment conditions (Figures S3 and S4), indicating that the observed changes are primarily due to regulatory effects on signaling and gene expression. Since CBD displayed only minimal regulatory effects, subsequent experiments and analyses focused on THC treatment alone with and without co-treatment with αTAn and αTAr.

### 3.2. Regulatory Effects of THC and Vitamin E on Expression of Genes Involved in Lipid Import

**Figure 2:**
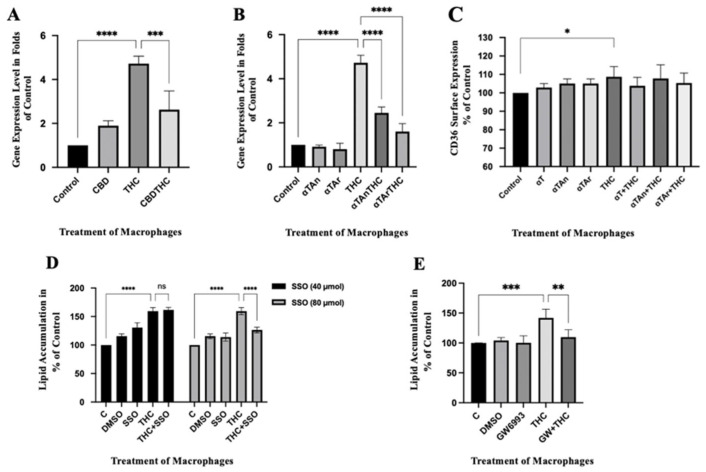
**Quantification of CD36 mRNA expression in THP-1 macrophages.** Cells were treated as indicated for 18 h (αTAr, αTAn (50 μM); THC, CBD (6 μM)). Total RNA was extracted, reverse transcribed, and gene expression measured by qPCR (Taqman). (**A**) Significant induction induced by THC was observed for CD36 gene expression at the mRNA level. CBD showed an inhibitory effect on CD36 gene expression when induced by THC. (**B**) Both αTAr and αTAn show inhibitory effects when induced by THC, and αTAr had a slightly stronger regulatory effect than αTAn. (**C**) THC significantly induced the surface expression of CD36 by FACS using ex488/em538 nm. (**D**) Staining with Nile Red, extraction with isopropanol, and measurement by FACS at ex561/em610 nm. Significant induction of lipid accumulation in THP-1 macrophages after 24 h of THC treatment and inhibition was observed by the CD36 inhibitor SSO (sulfo-N-succinimidyl oleate) at 80 μM. (**E**) Staining with Nile Red, extraction with isopropanol, and measurement by FACS at ex561/em610 nm. Significant induction of lipid accumulation in THP-1 macrophages after 24 h of THC treatment and inhibition was observed by PPAR*γ* inhibitor GW9662 (2-chloro-5-nitro-N-phenylbenzamide) at 10 μM (mean ± SEM, n = 6 for THC, CBD, αTAr, αTan, * *p* < 0.05, ** *p* < 0.005, *** *p* < 0.0005, **** *p* < 0.0001 relative to untreated control set to 100%; ns: non-significant).

To analyze the regulatory mechanisms involved in the observed lipid accumulation induced by THC and vitamin E analogs in THP-1 macrophages, we investigated the expression of the CD36/FAT scavenger receptor/fatty acid transporter (CD36/FAT) involved in lipid import at the mRNA level by RT-qPCR. THC significantly increased the expression of CD36/FAT, and co-treatment with CBD prevented it (Figure 2A). Co-treatment of THC with αTAn or αTAr inhibited CD36/FAT gene expression, and αTAr had a slightly stronger inhibitory effect when compared to αTAn and CBD (Figure 2B). Surface expression of CD36, as analyzed by FACS, was significantly induced by THC and reduced by αTAn and αTAr (Figure 2C). The covalent CD36 inhibitor, sulfo-N-succinimidyl oleate (SSO, 80 μM), and the PPARγ inhibitor (GW9662, 10 μM) both significantly inhibited lipid accumulation in THP-1 macrophages after treatment with THC (Figure 2D,E) [70].

### 3.3. Regulatory Effects of THC and Vitamin E on Expression of Genes Involved in Lipid Export

**Figure 3:**
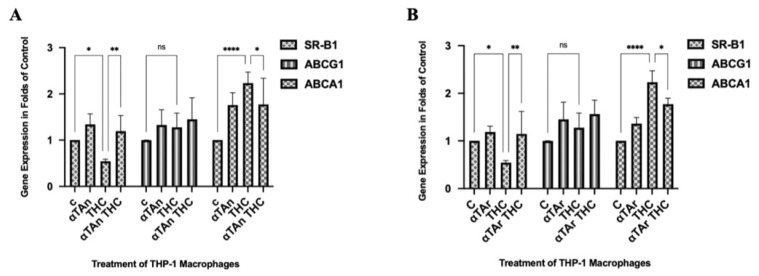
**Quantification of SR-B1, ABCA1, and ABCG1 mRNA expression in THP-1 macrophages.** Cells were treated as indicated for 18 h (αTAr, αTAn (50 μM); THC, CBD (6 μM)). Total RNA was extracted, reverse transcribed, and gene expression measured by qPCR (Taqman). (**A**) THC showed weak inhibitory effects on SR-B1 gene expression at the mRNA level, which was prevented by αTAn. No significant regulatory effects were observed with ABCG1 gene expression with THC and αTAn. THC significantly induced ABCA1 gene expression at the mRNA level, and co-treatment with αTAn showed inhibitory effects. (**B**) THC showed weak inhibitory effects on SR-B1 gene expression, which was prevented by αTAr. No significant regulatory effects were observed on ABCG1 gene expression with co-treatment with THC and αTAr. THC significantly induced ABCA1 gene expression at the mRNA level, and co-treatment with αTAr showed inhibitory effects. (mean ± SEM, n = 6 for THC, αTAr, αTAn * *p* < 0.05, ** *p* < 0.005, **** *p* < 0.0001 relative to untreated control set to 100%; ns: non-significant).

To further analyze the regulatory mechanisms that influence lipid accumulation in response to THC and αTAn or αTAr in human macrophages, we analyzed the expression of scavenger receptor B1 (SR-B1), ABC transporter A1 (ABCA1), and ABC transporter G1 (ABCG1), which are responsible for lipid and vitamin E export [71]. THP-1 macrophages were treated with THC with and without co-treatment with αTAn and αTAr for 18 h. A weak inhibitory effect on SR-B1 expression was observed with THC, which was prevented by αTAn (Figure 3A). No significant regulatory effect on ABCG1 expression was observed with THC, as well as with combined treatment with αTAn. Significant induction was measured with ABCA1 expression after THC treatment, and it was significantly inhibited with αTAn. A similar response on these genes was observed after co-treatment with αTAr (Figure 3B).

### 3.4. Regulatory Effects of THC and Vitamin E on Expression of Inflammatory Cytokines

**Figure 4:**
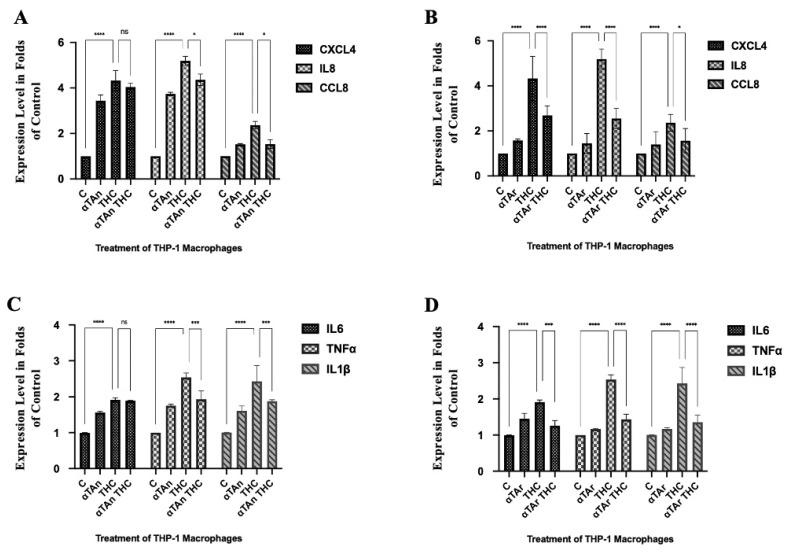
**Quantification of cytokine mRNA expression in THP-1 macrophages.** Cells were treated as indicated for 18 h (αTAr, αTAn (50 μM); THC, CBD (6 μM)). Total RNA was extracted, reverse transcribed, and gene expression measured by qPCR (Taqman). (**A**,**B**) Significant induction of CXCL4, IL8, and CCL8 gene expression was observed with THC, and it was prevented when co-treated with αTAn (**A**) or αTAr (**B**). (**C**,**D**) Significant induction of IL6, TNFα, and IL-1β was induced by THC, and it was prevented by co-treatment with αTAn (**C**) or αTAr (**D**). Co-treatment of THC with αTAr had a slightly stronger regulatory effect than co-treatment with αTAn. (mean ± SEM, n = 6 for THC, αTAr, αTAn * *p* < 0.05, *** *p* < 0.0005, **** *p* < 0.0001 relative to untreated control set to 100%; ns: non-significant).

To analyze whether THC and vitamin E have regulatory effects on inflammatory cytokines expression, we analyzed the expression of CXCL4/PF4, IL8/CXCL8, and CCL8/MCP-2 that mediate the pro-inflammatory response in macrophages [72,73,74]. THP-1 macrophages were treated with THC alone or together with αTAn and αTAr for 18 h. A significantly increased expression of CXCL8, IL8, and CCL8 was observed after treatment with THC (Figure 4A). An inhibitory effect was observed for IL8 and CCL8 after co-treatment with αTAn. A significant inhibitory effect was also observed for CXCL8, IL8, and CCL8 after co-treatment with αTAr (Figure 4B). Similar regulatory effects were observed for IL6, TNFα, and IL1β (Figure 4C,D). For most cytokines, αTAr showed a slightly stronger inhibitory effect when compared to αTAn.

### 3.5. Regulatory Effects of THC and Vitamin E on Cellular Cytokine Secretion

**Figure 5:**
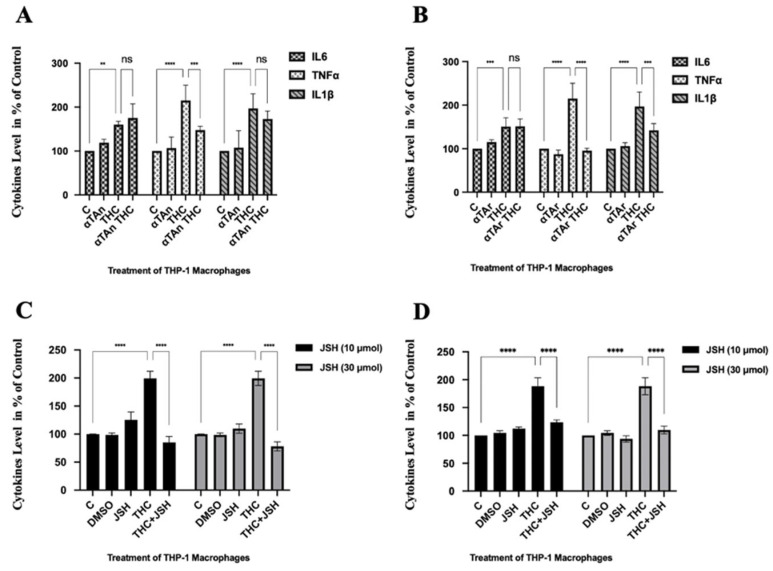
**Measurement of cytokine secretion in THP-1 macrophages.** THP-1 monocytes were differentiated with PMA (100 ng/mL) for 48 h and then treated with THC (6 μM), αTAn, and αTAr (50 μM) as indicated for 24 h. Secreted cytokines (IL6, TNFα, IL1β) were measured by ELISA using absorbance at OD 450 nm (**A**). Significant induction was observed for IL6, TNFα, and IL1β with THC, which was prevented by co-treatment with αTAn only for TNFα. (**B**) Significant induction was observed for IL6, TNFα, and IL1β with THC, which was prevented by co-treatment with αTAr only for TNFα and IL1β. Co-treatment of THC with αTAr had a slightly stronger regulatory effect than co-treatment with αTAn. (**C**) Significant induction was observed for TNFα (**C**) and IL1β (**D**) with THC, which was prevented by co-treatment with NF*κ*B inhibitor JSH-23 at both 10 and 30 μM. (mean ± SEM, n = 6 for THC, αTAr, αTAn ** *p* < 0.005, *** *p* < 0.0005, **** *p* < 0.0001 relative to untreated control set to 100%; ns: non-significant).

To analyze whether the regulatory effects on cytokine mRNA expression lead to altered levels of secreted cytokine proteins, which are relevant to inflammation, cell supernatants were collected, and the levels of a few regulated cytokines were measured by ELISA. Significantly increased levels of the secreted pro-inflammatory cytokines IL6, TNFα, and IL1β were observed after treatment with THC (Figure 5A,B). However, no significant inhibitory effect was observed with IL6 and IL1β after co-treatment with αTAn, and only a weak regulatory effect was observed with TNFα (Figure 5A). No inhibitory effect was observed with IL6 expression after co-treatment with αTAr. However, a significant inhibitory effect was observed with TNFα and IL1β expression after co-treatment with αTAr (Figure 5B). For these cytokines, αTAr showed a slightly stronger inhibitory effect when compared to αTAn. Co-treatment with the NF*κ*B inhibitor JSH-23 reduced the induction of TNF*α* and IL1β after induction by THC (Figure 5C,D) [75]. Whereas overall the regulatory pattern of THC, αTAn, and αTAr was similar at the mRNA and secreted cytokines level, observed small differences may be the result of posttranscriptional and posttranslational effects, such as differential cytokine maturation/cleavage and secretion.

### 3.6. Regulatory Effects of THC and Vitamin E on Reactive Oxygen Species (ROS) in THP-1 Macrophages

**Figure 6:**
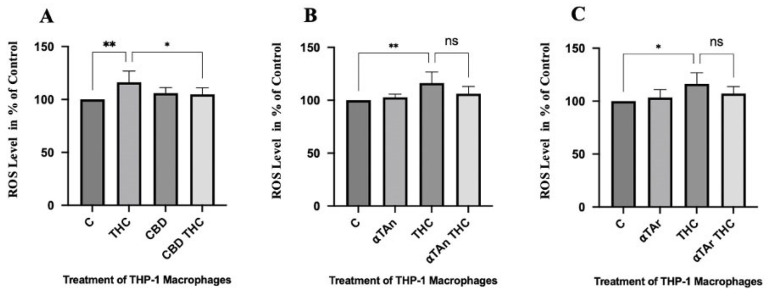
**Regulation of ROS production in THP-1 macrophages.** THP-1 monocytes were differentiated with PMA (100 ng/mL) for 48 h and then treated with THC (6 µM), αTAn, and αTAr (50 µM) as indicated for 24 h. The results were analyzed by FACS using ex488/em538 nm. (**A**) Significant induction of ROS production was observed with THC, which was prevented by co-treatment with CBD. (**B**,**C**) Only non-significant inhibitory effects were observed by co-treatment with αTAn (**B**) or αTAr (**C**). (mean ± SEM, n = 6 for THC, CBD, αTAr, αTAn * *p* < 0.05, ** *p* < 0.005 relative to untreated control set to 100%; ns: non-significant).

THC has previously been reported to increase the production of reactive oxygen species (ROS) that may contribute to the observed regulatory effects [76,77]. Thus, we measured free radical production in THP-1 macrophages after treatment with THC, CBD, αTAn, and αTAr. THC significantly increased ROS production in THP-1 macrophages, and co-treatment with CBD prevented it (Figure 6A). However, co-treatment with either αTAn or αTAr had only a weak inhibitory effect on ROS production induced by THC, suggesting that they are insufficiently converted into αT and therefore not completely able to act as chemical antioxidants in this experimental setting (Figure 6B,C). Since activation of CB1 can increase ROS production, αTAn and αTAr may also reduce ROS by binding to and inhibiting CB1 via non-antioxidant mechanisms [77].

### 3.7. Regulatory Effects of THC and Vitamin E on CD36 Activation via CB1

**Figure 7:**
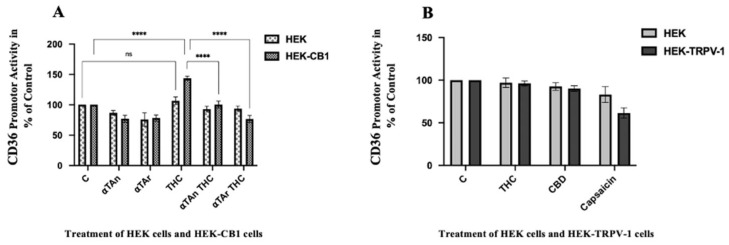
**Quantification of CD36 Promoter Activities via CB1 and TRPV-1 in HEK293 cells.** HEK293 and HEK-CB1 cells were transfected with reporter plasmids one day before treatment. HEK293 and HEK-CB1 cells were treated with THC (6 μM), αTAn, and αTAr (50 μM) as indicated for 24 h. Dual luciferase assays were measured using a microtiter plate reader (Clariostar, BMG Labtech). (**A**) Significant induction of CD36 promoter activities was observed in HEK-CB1 with THC, which was prevented by co-treatment with αTAn and αTAr. (**B**) Neither THC nor CBD had regulatory effects in HEK-TRPV-1 cells, but control capsaicin (6 μM) was inhibitory. (mean ± SEM, n = 6 for THC, αTAr, aTAn **** *p* < 0.0001 relative to untreated control set to 100%; ns: non-significant).

THC and CBD bind and activate cannabinoid receptors, such as CB1, CB2, or TRPV-1. When testing the mRNA expression levels of these receptors during differentiation of monocytes to macrophages for 48 h, CB2 was not expressed, and TRPV-1 and CB1 were expressed and constant (Figure S5). To determine the possible involvement of these receptors in the observed regulatory effects, we transfected HEK293 cells overexpressing cannabinoid receptors (CB1 or TRPV-1) with a luciferase reporter plasmid controlled by the human CD36 promoter. In HEK-CB1, we observed a significant induction of the CD36 promoter activity via CB1 after THC treatment (Figure 7A), but not in HEK-TRPV-1 via TRPV-1 (Figure 7B), albeit capsaicin, a natural ligand of TRPV-1, had an inhibitory effect. Accordingly, TRPV-1 was reported not to be regulated by THC [78]. Both αTAn and αTAr showed significant inhibitory effects on CD36 promoter activities in HEK-CB1 cells after induction by THC through CB1.

### 3.8. Regulatory Effects of THC and Vitamin E on CB1 Activation

**Figure 8:**
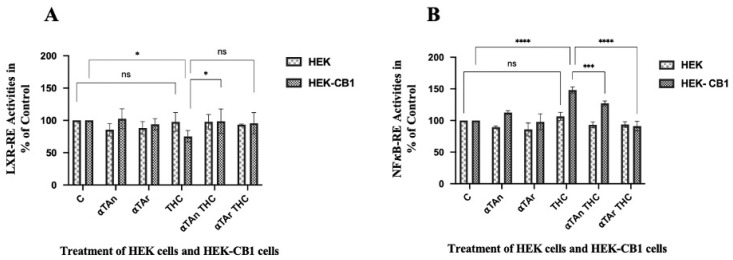
**Quantification of LXR and NFkB transcription factor activities via CB1 in HEK293 cells.** HEK293 and HEK-CB1 cells were transfected with reporter plasmids one day before treatment. HEK and HEK-CB1 cells were treated with THC (6 μM), αTAn, and αTAr (50 μM) as indicated for 24 h. Dual luciferase assays were measured using a microtiter plate reader (Clariostar, BMG Labtech). (**A**) Weak inhibitory effects on LXR-RE-reporter were observed in HEK-CB1 cells with THC that were prevented by αTAn and αTAr. (**B**) Significant induction of NF*κ*B-RE-reporter was observed after THC treatment, which was prevented by αTAn and more so by αTAr. (mean ± SEM, n = 6 for THC, αTAr, αTAn * *p* < 0.05, *** *p* < 0.0005, **** *p* < 0.0001 relative to untreated control set to 100%; ns: non-significant).

To delineate which CD36 promoter element is involved in its induction by THC in HEK-CB1 cells, we transfected these cells with luciferase reporter plasmids controlled by response elements relevant for its regulation (e.g., LXR-RE, Nrf2-RE, NFκB-RE, and PPARγ-RE). First, the expression of these transcription factors during THP-1 differentiation was checked by RT-qPCR. When PPARγ expression was increased [45], the expression of LXRα and LXRβ remained constant (Figure S5). We performed dual luciferase analysis of the HEK293 and HEK-CB1 cells after transfection of reporter plasmids for these transcription factors. Vitamin E was able to inhibit NFκB activity via the CB1 receptor, which may also be related to the regulatory effects observed for oxidative stress (ROS production) and inflammatory cytokines regulation. Only weak regulatory effects were observed with the LXR-RE reporter vector, and THC was inhibitory (Figure 8A). In cells overexpressing CB1, we observed activation of NFκB by THC that was inhibited by αTAn and more so by αTAr (Figure 8B).

### 3.9. Regulatory Effects of THC and Vitamin E on TRPV-1 Activation

**Figure 9:**
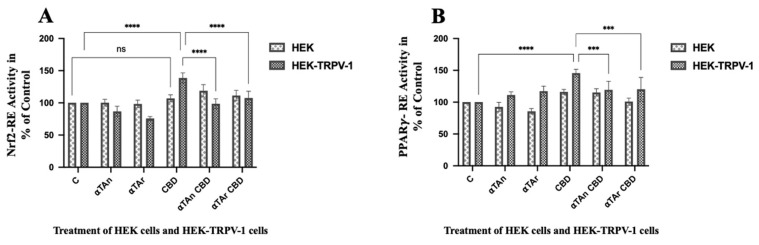
**Quantification of Nrf2 and PPARγ transcription factor activities via TRPV-1 in HEK293 cells.** HEK293 and HEK-TRPV-1 were transfected with reporter plasmids one day before treatment. HEK and HEK-TRPV-1 cells were treated with CBD (6 μM), αTAn, and αTAr (50 μM) as indicated for 24 h. Dual luciferase assays were measured using a microtiter plate reader (Clariostar, BMG Labtech). (**A**) Significant induction of the Nrf2-RE reporter was observed in HEK-TRPV-1 with CBD, which was prevented by co-treatment with αTAn and αTAr. (**B**) Significant induction of PPARγ-RE was observed after treatment with CBD, which was prevented by αTAn and αTAr. (mean ± SEM, n = 6 for CBD, αTAr, αTAn *** *p* < 0.0005, **** *p* < 0.0001 relative to untreated control set to 100%; ns: non-significant).

THC and CBD did not affect CD36 promoter activity in HEK293 and HEK-TRPV-1 cells (Figure 7B). Thus, TRPV-1 may not be involved in the regulatory effects of THC on lipid accumulation as a result of up-regulation of CD36, but it may trigger other cellular responses. Since TRPV-1 is not regulated by THC, we tested for potential regulatory effects of CBD. In HEK-TRPV-1 cells, CBD activated Nrf2-RE and PPARγ-RE, and αTAn and αTAr reduced it (Figure 9A,B). Nuclear translocation and activation of Nrf2 by CBD have been previously observed in glioblastoma cells, whereas co-treatment with vitamin E inhibited it, but the mechanisms are complex and need to be further evaluated [79]. Further, molecular modeling suggested vitamin E as a potential ligand of Nrf2, and it remains to be shown whether αTA can do the same [80]. The expression of protein phosphatase 2A (PP2A), which has been associated with the formation of foam cells, was slightly affected by THC, αTAn, αTAr, and CBD, but the regulatory effects were weak and non-significant (Figure S6) [81].

## 4. Discussion

Cannabinoid receptors and their ligands have emerged as important regulators of metabolic diseases such as dyslipidemia, obesity, and atherosclerosis [82]. CB1 is primarily expressed in the nervous system but also in peripheral tissues and immune cells, including monocytes/macrophages. In contrast, CB2 expression is restricted to peripheral tissues and immune cells (B cells, T cells, and monocytes/macrophages), but it can also be activated during inflammation [83]. At the cellular level, over-activation of these receptors by natural and synthetic cannabinoids can induce the expression of CD36/FAT, involving activation of PPARγ, and the higher cellular uptake of lipids into cells is thought to increase the risk for obesity and the metabolic syndrome [44,45,84,85,86].

In addition to increasing lipid accumulation, the natural CB1 receptor agonist, THC, can induce cellular oxidative stress, proinflammatory cytokines and chemokines production, increase monocyte adhesion, and decrease the expression of antioxidant-related genes through activation of p38 mitogen-activated protein (MAP) kinase and NFκB pathways [76]. Accordingly, CB1 activation leads to stimulation of the MAP kinase pathway, which causes oxidative stress, inflammation, and cell death in human coronary artery endothelial cells [77,87].

Vitamin E is the major fat-soluble antioxidant and is naturally present as eight different analogs, α-, β-, γ, δ-tocopherols and tocotrienols, all with an *RRR* side chain conformation. As recently discussed, only natural *RRR*-αT and synthetic *2R*-αT have all the activities to prevent vitamin E deficiency syndromes and therefore should be called vitamin E [88]. To prevent oxidation and increase the shelf life of supplements, infant formula, and cosmetics, natural and synthetic αT is often stabilized by modifying the hydroxy group with an acetate to form α-tocopheryl acetate (αTAn, αTAr), converting a weakly acidic molecule into a neutral ester. Upon ingestion, αTA is efficiently converted to natural αT and therefore acts as a precursor of αT. However, incomplete conversion may affect the bioavailability of natural and synthetic αTA [24,25]. When in matrices during digestion and in patients with severe cholestasis and intestinal genetic hypocholesterolemia, hydrolysis and uptake of αTA is reduced when compared to mixed micelles [35,89]. In cultured intestinal epithelial cells (Caco-2), some intact αTAr can be absorbed, transported, and secreted to the basolateral side [90,91].

Likewise, conversion of αTA to αT is not observed on the surface or in the horny layers of human skin, while up to 50% is converted in the underlying skin [31,36]. In the skin, topical treatment with αTA improved a number of dermatologic conditions [92]. Increased levels of intact αTA are reportedly detected upon inhaling aerosols containing αTA, and together with THC, are associated with cases of electronic-cigarette or vaping product use-associated lung injury (EVALI) [27,29,30,93,94,95,96,97,98,99,100]. In these studies, an increased number of macrophage foam cells was detected in bronchoalveolar lavages and lungs after inhaling e-cigarette aerosols from illicit products containing THC and αTA, which were present as vape-cartridge additives [28,29,30]. Thus, αTA in lungs may act as intact αTA, and it is not clear whether it has similar regulatory effects as αT, whether and to what degree it is converted to αT, and whether it can affect lipid accumulation, inflammation, signaling, and gene expression. In the lungs, aggregate formation by αTA with THC and enhanced solubilization may dysregulate signaling receptors such as CB1 and CB2, triggering lipid accumulation [5,101]. Further, the regulatory effects of intact αTA were reported to maintain mesenchymal stem and progenitor cells in their primitive state and to attenuate mitochondrial oxygen consumption by mimicking hypoxia [26].

In the experimental conditions used in our study with cultured THP-1 macrophages, intact αTA remains present in the culture medium for prolonged periods. The acetate moiety may influence not only its antioxidant capacity but also its cellular uptake and interactions with membranes and cell surface receptors. Further, the altered water solubility and stability of αTA when compared to αT may offer more ways to interact with membranes and cellular receptors (e.g., via CB1/CB2, TRPV-1, CD36, and SR-B1) and to change membrane properties by acting as a dipole or a linactant [5,6,7,27,28,101,102,103]. Differential effects of αTAn and αTAr can be explained by αTAr having a more flexible hydrophobic side chain able to interact with receptors and membranes in eight times more ways when compared to αTAn, not only resulting in differential signaling and gene expression but also in conversion to metabolites with stereoisomer-specific bioactivity.

**Figure 10:**
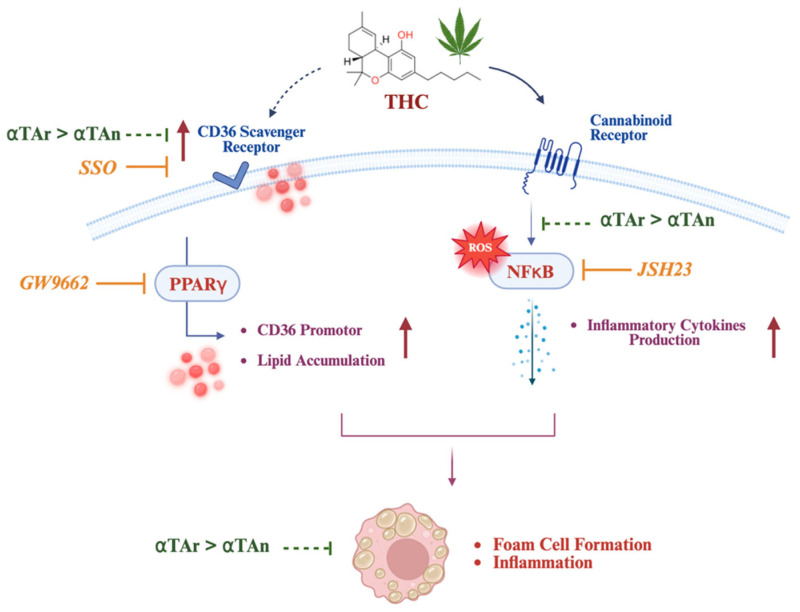
**Foam cell formation as a result of an imbalance of lipid homeostasis.** This schematic illustrates the hypothetical mechanisms by which THC affects foam cell formation through modulation of lipid uptake and inflammatory signaling pathways. THC interacts with the cannabinoid receptor (CB1), leading to activation of the NFκB pathway and increased ROS production. Activated NFκB stimulates production of inflammatory cytokines, further contributing to foam cell formation. This inflammatory pathway is suppressed by the NFκB inhibitor JSH-23. Activation of the CD36 scavenger receptor increases lipid accumulation within the cell after THC treatment and contributes to foam cell formation. This pathway is inhibited by SSO, a CD36 inhibitor. THC also regulates PPARγ, which enhances CD36 promoter activity and further promotes lipid uptake; the PPARγ inhibitor GW9662 attenuates this effect. Overall, αTA showed inhibitory effects on CD36 gene regulation, inflammatory cytokine production via NFκB activation, and foam cell formation. αTAr has a stronger effect than αTAn, suggesting differential protective effects against THC-induced lipid accumulation and inflammation.

We observed increased levels of lipid accumulation in THP-1 macrophages after treatments with THC, which could be reduced by co-treatment with αTAn, αTAr, and to a lesser extent by CBD (Figure 10). The involvement of CD36 is suggested by increased surface expression after THC treatment and reduced lipid accumulation after inhibition by SSO. As a possible mechanism, we observed increased levels of ROS after THC treatment, suggesting an imbalance between oxidants and antioxidants; however, ROS levels were reduced by αTAn, αTAr, or CBD only partially. These results suggest that upon uptake into macrophages, αTA may only be partially converted to αT, insufficient to prevent lipid peroxidation and cellular lipotoxicity. Interestingly, whereas αTAn and αTAr showed similar effects in preventing ROS production, αTAr was generally more efficient in reducing signaling and gene expression in response to THC. This may suggest that αTAn and αTAr interact differently with membranes and signaling receptors/enzymes, or possibly they are taken up and metabolized in a stereospecific manner.

Further, we observed that genes involved in lipid import (CD36/FAT) and export (SR-B1, ABCA1) are differently regulated, possibly generating an imbalance of lipid flux in macrophages with a net effect of accumulation of lipids and foam cell formation (Figure 10). αTAn and αTAr both inhibited ABCA1 expression after stimulation with THC. Inhibitory effects of *RRR*-αT and *RRR*-γT on ABCA1 expression have been previously described, and they were mediated by LXR [104]. In addition to ABCA1/G1, other transporters may be involved in lipid export, such as the multidrug transporter, P-glycoprotein (ABC transporter of subfamily B (ABCB1)) [102,105]. THC significantly induced the expression of inflammatory cytokines at both the mRNA and protein levels. Stronger anti-inflammatory effects were measured with αTAr when compared to αTAn after induction by THC, both when measured at the mRNA level and as secreted cytokines.

To delineate the signaling pathways involved in the induction of CD36/FAT and its inhibition by αTA, we transfected a CD36 promoter–luciferase reporter plasmid into cells that overexpress CB1 or TRPV-1. Although HEK-CB1 and HEK-TRPV-1 cells do not accumulate lipids, and receptor overexpression may not reflect the physiological situation present in THP-1 macrophages, they are a useful tool to delineate the receptors involved in signaling by THC and αTA. These results suggested that CD36 expression is activated by THC via CB1 and not via TRPV-1, in line with TRPV-1 not being responsive to THC [78]. Amongst the tested transcription factors involved in regulating CD36, THC had no effect on PPARγ activity in HEK-TRPV-1 but inhibited via LXR, possibly explaining the inhibition of SR-B1 observed at the mRNA level [106]. Nevertheless, in THP-1 macrophages, the PPARγ inhibitor GW9662 efficiently prevented lipid accumulation, in line with findings of increased PPARγ activity after CB1 activation by Win55,212-2 [45]. NFκB was activated by THC in HEK-CB1, and the NFκB inhibitor JSH23 reduced cytokines in THP-1 macrophages, suggesting that inflammatory cytokine expression may be induced by THC via activation of CB1, and with αTAr being more effective than αTAn. CD36 has also been identified as a potential target of NFκB, but a major response element in the CD36 promoter remains to be located [107]. NFκB is activated by oxLDL via CD36 [108], and vitamin E analogs differently inhibit NFκB expression [109]. At the present time, we cannot exclude that additional receptors responsive to cannabinoids may play a role in the observed regulatory effects. We can exclude CB2 since it was not expressed during differentiation of THP-1 cells to macrophages, and GPR55 expression remained low, suggesting it is less involved, at least in our experimental system. In other cells, GPR55 was reported to modulate lipid homeostasis, atherosclerosis, and inflammation in response to its ligands by affecting the expression of CD36/FAT and ABCA1/G1 and of inflammatory cytokines [84]. Further, a selective agonist of GPR55, O-1602, which does not activate CB1/CB2, also increased macrophage lipid accumulation and foam cell formation, events that could be inhibited by the antagonist cannabidiol [84].

Interference with increased levels of lipids and ROS, leading to lipid peroxidation, is considered a preventive and therapeutic approach for inflammatory, cardiovascular, and liver diseases. For vitamin E, the best evidence for disease preventive effects has been reported for diseases that involve an inflammatory and lipid-mediated component, such as non-alcoholic steatohepatitis (NASH, now called metabolic dysfunction-associated steatohepatitis (MASH)), cardiovascular disease, arthritis, and some cancers [109,110,111,112,113,114,115,116]. In line with previous research [45,84], we observed excessive cellular intake of lipids (imported by CD36/FAT) after THC treatments, and the increased levels of ROS suggest that this may involve increased levels of toxic lipid species such as ceramides and diacylglycerols [117]. It is reported in the literature that in diet-induced obese mice, inhibition of CD36/FAT in the liver was achieved by down-regulation of peripheral CB1 by antisense oligonucleotide and resulted in improved insulin sensitivity, glucose homeostasis, and liver steatosis [86]. In obese db/db mice, pharmacological blockade of CB1 improved hyperlipidemia and hyperinsulinemia, and it increased inflammatory cytokine-mediated NASH development [118]. Accordingly, in addition to having reduced appetite, CB1^−/−^ mice had altered peripheral energy metabolism, protecting them from high-fat diet-induced weight gain or lipid deposition [8,9]. From studies in zebrafish and rats, vitamin E deficiency was suggested to dysregulate energy metabolism [119,120], but a possible involvement of CB1 remains to be investigated. Interestingly, knockdown or inhibition of the CB1 receptor increased the risk for neurodevelopmental disorders (e.g., autism spectrum disorder-like), and CB1 receptor ligands and vitamin E have been suggested to act as modulators of these disorders [121,122,123,124]. Whereas in healthy subjects, αTA is generally completely converted to αT during uptake, inefficient hydrolysis of αTA may occur in patients with exocrine pancreatic insufficiency (e.g., cystic fibrosis, preterm infants) and interfere with signaling by cannabinoid receptors [125,126,127]. Further, when injected as an adjuvant, αTA may stay intact and inhibit CB2, possibly explaining booster effects observed with the inactivated vaccine for Newcastle disease virus in chickens and with antigen-specific antibody responses in mice [128,129].

## 5. Conclusions

The findings of this study provide mechanistic insights into how alterations in lipid homeostasis induced by THC through CB1 receptor activation contribute to inflammation. The results demonstrate that these pro-inflammatory lipid changes can be differentially mitigated by vitamin E analogs and cannabidiol (CBD). It is conceivable that increasing the bioavailability of vitamin E analogs using targeted nanoparticles may be a strategy to reduce the expression of inflammatory cytokines and accumulation of lipids in macrophages, but it may also inadvertently affect signaling and gene expression essential for immune and neurodevelopmental processes [74]. Whether the vitamin E analogs similarly modulate the action of endocannabinoids within the vascular, central, and peripheral nervous systems and alleviate neuropathic and chronic inflammatory pain and other events needs to be further investigated [130,131,132]. Future investigations should focus on the therapeutic potential of cannabinoids and vitamin E analogs in diseases characterized by concurrent inflammation and lipid metabolic dysregulation.

## Data Availability

The original contributions presented in this study are included in the article/Supplementary Materials. Further inquiries can be directed to the corresponding authors.

## Supplementary Materials

The following supporting information can be downloaded at: https://www.mdpi.com/article/10.3390/antiox15010119/s1, Table S1: TaqMan Probes; Figure S1: Structures of Vitamin E analogues; Figure S2: Luciferase assay procedure; Figure S3: Cell cycle analysis; Figure S4: Cytotoxicity assay with THP-1 monocytes as measured with Lactate dehydrogenase (LDH) release assay; Figure S5: Differentiation of THP-1 monocytes to macrophages; Figure S6: Quantification of PP2AC mRNA expression in THP-1 macrophages.

## Abbreviations

The following abbreviations are used in this manuscript:

ABCA1	ATP-Binding Cassette Transporter A1
ABCB1	ATP-Binding Cassette Transporter B1 (P-glycoprotein)
ABCG1	ATP-Binding Cassette Transporter, subfamily G, member 1
ATCC	American Type Culture Collection
CB1	Cannabinoid Receptor type 1
CB2	Cannabinoid Receptor type 2
CBD	Cannabidiol
CCL8	C-C motif chemokine ligand 8 (MCP2)
CD36/FAT	Cluster of Differentiation 36/Fatty Acids Transporter
CXCL4	Platelet Factor 4
CXCL8	Interleukin-8
DCF	Dichlorofluorescein
ELISA	Enzyme-linked Immunosorbent Assay
FABP4	Fatty Acid-Binding Protein 4
FABP5	Fatty Acid-Binding Protein 5
FACS	Fluorescence-Activated Cell Sorting
FBS	Fetal bovine serum
GAPDH	Glyceraldehyde-3-phosphate dehydrogenase
IL1β	Interleukin-1 beta
IL6	Interleukin-6
LDH	Lactate Dehydrogenase
LXR	Liver X Receptor
MAP Kinase	Mitogen-Activated Protein Kinase
MASH	Metabolic Dysfunction-Associated Steatohepatitis
NASH	Non-Alcoholic Steatohepatitis
NFκB	Nuclear Factor kappa B
Nrf2	Nuclear Factor Erythroid 2-related Factor 2
PI3K	Phosphoinositide 3-Kinase
PMA	Phorbol 12-Myristate 13-Acetate
PPARγ	Peroxisome Proliferator-Activated Receptor gamma
PP2A	Protein Phosphatase 2A
ROS	Reactive Oxygen Species
SR-B1	Scavenger Receptor Class B Type 1
SSO	Sulfosuccinimidyl oleate
αT	Alpha-Tocopherol
αTA	Alpha-Tocopherol Acetate (αTAn or αTAr)
αTAn	Natural alpha-Tocopheryl Acetate
αTAr	Synthetic *all-rac*-alpha-Tocopheryl Acetate
THC	Tetrahydrocannabinol
TNFα	Tumor Necrosis Factor alpha
αTP	Alpha-Tocopheryl Phosphate
TRPV-1	Transient Receptor Potential Vanilloid 1
