# Supplementary material for: Differential Regulatory Effects of Cannabinoids and Vitamin E Analogs on Cellular Lipid Homeostasis and Inflammation in Human Macrophages
Source: Antioxidants (Basel). 2026 Jan 16;15(1):119. doi: 10.3390/antiox15010119 (PMC12837758; doi:10.3390/antiox15010119)
Supplement: Supplementary file 1 [file antioxidants-15-00119-s001.zip › antioxidants-4039870-supplementary.pdf]

Supplementary Information to:

Differential Regulatory Effects of Cannabinoids and Vitamin E Analogs on Cellular Lipid Homeostasis and Inflammation in Human Macrophages

Mengrui Li[1,2], Sapna Deo[1,2,3], Sylvia Daunert[1,2,3*], Jean-Marc Zingg[1,2*]

[1]Department of Biochemistry and Molecular Biology, Miller School of Medicine, University of Miami, Miami, FL 33136-6129, USA

[2]Dr. John. T. Macdonald Foundation Biomedical Nanotechnology Institute, University of Miami, Miami, FL 33146-2101, USA

[3]University of Miami Clinical and Translational Science Institute, University of Miami, Miami, FL 33136-6129, USA

*corresponding authors

Gene	Product Name
ABCA1	Hs01059137_m1
ABCG1	Hs00245154_m1
CB1	Hs00275634_m1
CB2	Hs05019229_s1
CCL8	Hs04187715_m1
CD36	Hs01567191_m1
CXCL4	Hs00427220_g1
CXCL8 (IL8)	Hs00174103_m1
GAPDH	Hs02786624_g1
GPR55	Hs00271662_s1
IL6	Hs00174131_m1
IL1β	Hs01555410_m1
IL10	Hs00961622_m1
LXR	Hs01027215_g1
PPARγ	Hs01115513_m1
SR-B1	Hs00969821_m1
TNFα	Hs00174128_m1
TRPV1	Hs00218912_m1

Table S1. TaqMan Probes.

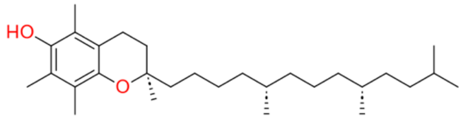

αT (Natural *RRR*-α-Tocopherol)

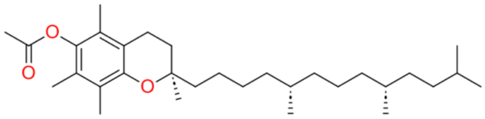

αTAn (Natural *RRR*-α-Tocopheryl Acetate)

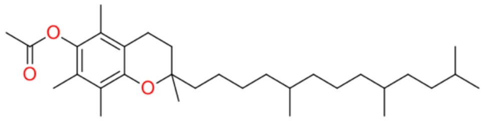

αTAr (Synthetic racemic *all-rac*-α-Tocopheryl Acetate)

Figure S1. Structures of Vitamin E analogs.

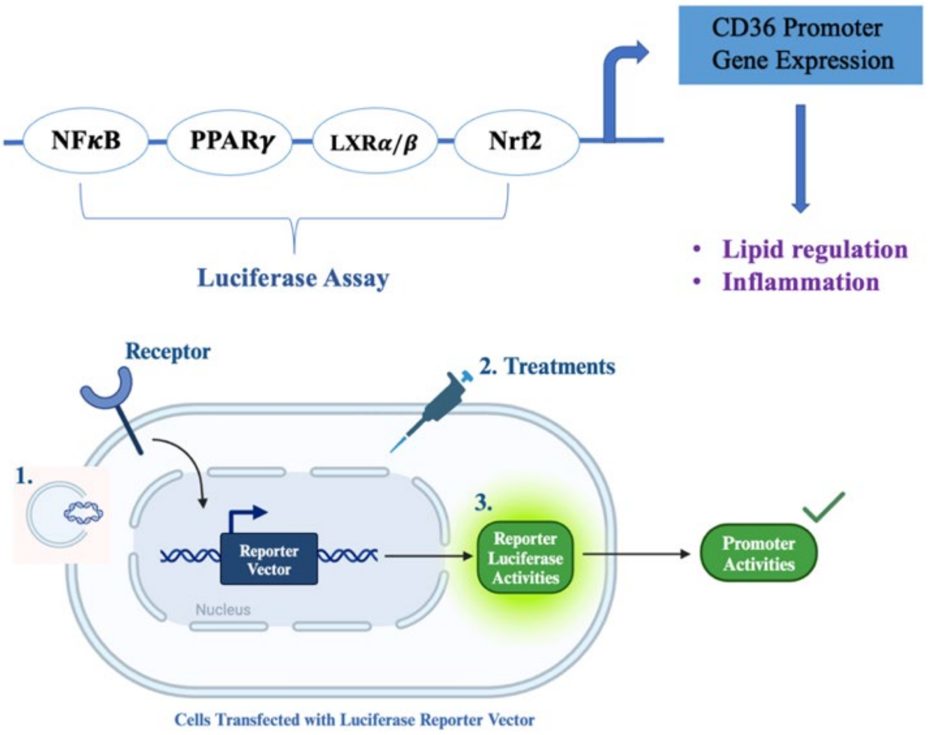

Figure S2. Luciferase assay procedure.

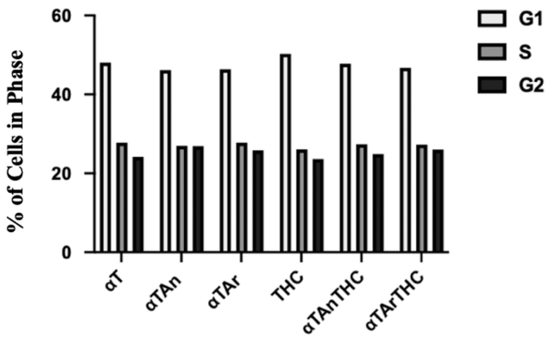

Figure S3. Cell cycle analysis. Cell cycle analysis after treatment with αT, αTAn, αTAr (50 µM) with/without THC or CBD (6 µM), for 18 h as assessed by propidium iodide staining (% cells in each phase, mean ± SEM, n = 4, total set to 100%). A small increase in G1 with THC but not significant.

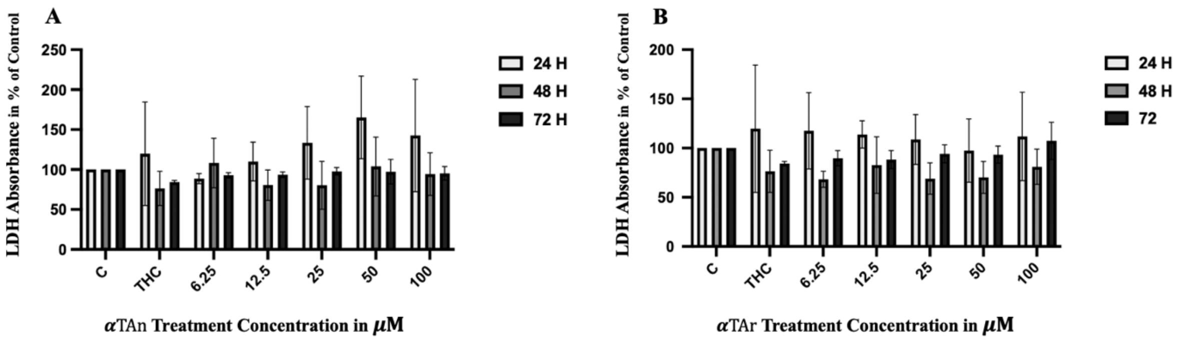

Figure S4. Cytotoxicity assay with THP-1 monocytes as measured with Lactate dehydrogenase (LDH) release assay. (A). Cells were treated with αTAn or αTAr at the indicated concentrations (0-100 μM), with THC (6 μM) for 24 h. LDH release was measured at OD590 (mean ± SEM, n = 4 for THC, relative to untreated control set to 100%). Increased LDH release was only observed at 48 h at 50 and 100 μM αTAn. THC reduced the LDH release, most likely as result of inhibition of cell proliferation**. (B).** There is no significantly increased LDH release with combined THC and αTAr treatment in THP-1 macrophages with both 24h, 48h and 72h.

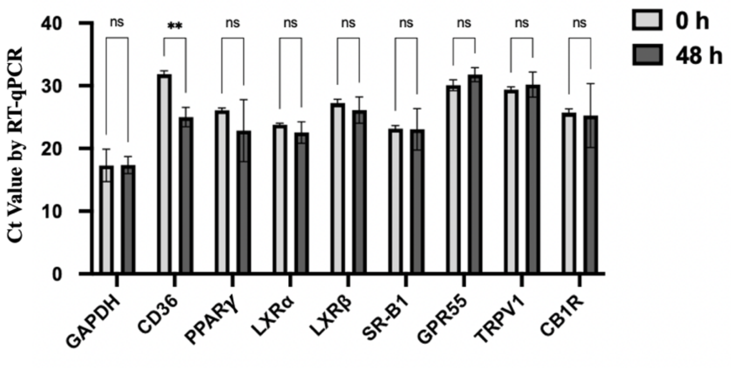

Figure S5. Differentiation of THP-1 monocytes to macrophages. THP-1 monocytes were differentiated by PMA (phorbol 12-myristate 13-acet ate) treatment (100 nmol) of 0 h and 48 h. There is a significant induction of CD36 gene expression observed in macrophages after 48 h PMA treatment. No significant effect was found on expression of the other genes with 0 and 48 h PMA treatment. CB2R was not detectable. ** $p < 0.005$; ns: non-significant.

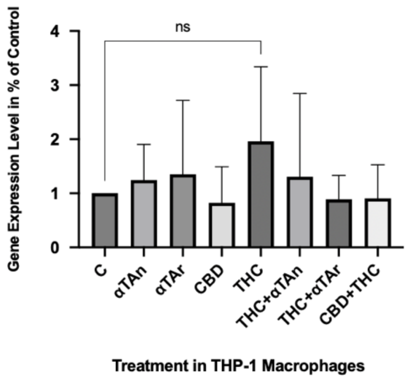

Figure S6. Quantification of PP2AC mRNA expression in THP-1 macrophages. In THP-1 macrophages, non-significant induction was observed with THC that was reduced by co-treatment with αTAn, αTAr and CBD. Cells were treated as indicated for 18 h (αTAr, αTAn (50 µM); THC, CBD (6 µM)). PP2AC was measured using quantitative RT-PCR with Taqman probes (n=6, $p < 0.05$, compared to control, ns: non-significant).
